# Efficacy of ultrasound-guided technique for radial artery catheterization in pediatric populations: a systematic review and meta-analysis of randomized controlled trials

**DOI:** 10.1186/s13054-020-02920-8

**Published:** 2020-05-06

**Authors:** Wen Zhang, Kunpeng Li, Hui Xu, Dawei Luo, Changbin Ji, Keshi Yang, Qinghua Zhao

**Affiliations:** 1 Department of Ultrasound, Liaocheng People’s Hospital, Liaocheng City, Shandong Province China; 2 Department of Orthopaedics, Liaocheng People’s Hospital, No 67 Dongchang West Road, Liaocheng City, 252000 Shandong Province China

**Keywords:** Ultrasound, Palpation, Arterial catheterization, Pediatric populations, First-attempt

## Abstract

**Background:**

The use of an ultrasound-guided technique for radial arterial catheterization has not been well established in pediatric patients. We conducted a systematic review and meta-analysis to evaluate the efficacy of the ultrasound-guided technique for radial artery catheterization in pediatric populations.

**Method:**

A systematic review of PubMed, Medline, Embase, and the Cochrane library was performed from their date of inception to December 2019. In this meta-analysis, we conducted online searches using the search terms “ultrasonography,” “ultrasonics,” “ultrasound-guided,” “ultrasound,” “radial artery,” “radial arterial,” “catheter,” “cannula,” and “catheterization.” The rate of the first-attempt and total success, mean attempts to success, mean time to success, and incidence of complications (hematomas) were extracted. Data analysis was performed with RevMan 5.3.5.

**Results:**

From 7 relevant studies, 558 radial artery catheterizations were enrolled, including 274 ultrasound-guided and 284 palpation catheterizations. The ultrasound-guided technique could significantly improve the rate of first-attempt and total success (RR 1.78, 95% CI 1.46 to 2.18, *P* < 0.00001; RR 1.33; 95% CI 1.20 to 1.48; *P* < 0.00001). However, there was significant heterogeneity for the total success rate among the included studies (*I*^2^ = 67%). The ultrasound-guided radial artery catheterization was also associated with less mean attempts and mean time to success (WMD − 1.13, 95% CI − 1.58 to − 0.69; WMD − 72.97 s, 95% CI − 134.41 to − 11.52) and lower incidence of the hematomas (RR 0.17, 95% CI 0.07 to 0.41).

**Conclusions:**

The use of the ultrasound-guided technique could improve the success rate of radial arterial catheterization and reduce the incidence of hematomas in pediatric patients. However, the results should be interpreted cautiously due to the heterogeneity among the studies.

## Background

Arterial catheterization is a common and essential procedure performed in many clinical settings, such as the emergency department, intensive care unit, and operating room [1–3]. It allowed continuous blood pressure monitoring and repeated arterial blood sampling. The radial artery is the most common site for arterial catheterization because of its superficial location, dual arterial supply to the hand, and low rate of complications [4, 5]. Traditionally, the radial artery catheterization is performed by the guidance of anatomical knowledge and pulse palpation. However, a traditional palpation technique can be technically challenging, often requiring multiple attempts and causing patient discomfort and suffering, particularly in pediatric patients or patients with hypotension, edema, and obesity [6, 7].

An ultrasound-guided technique has been commonly used as a good tool for central vein catheterization with the development of ultrasound applications in medicine. A series of studies [8] have confirmed that the use of ultrasound guidance could increase the success rates and reduce the rates of complications, as compared with the traditional palpation technique. With respect to radial arterial catheterization, previous systematic reviews and meta-analyses [9–11] comparing the ultrasound-guided technique versus the traditional palpation have reported higher first-pass success rates, less time to catheter insertion, and fewer hematomas with ultrasound-guided radial artery access, although several pediatric studies were included in these analyses [12, 13]. However, the use of ultrasound guidance for radial arterial catheterization in pediatric populations has not been well established. A recent systematic review and meta-analysis on arterial cannulation in pediatrics conducted by Aouad-Maroun [14] yielded limited results because they include all arterial cannulation (radial, ulnar, brachial, femoral, or dorsalis pedis artery). Since then, there are two more randomized controlled trials (RCTs) published on this topic. With accumulating evidence, we therefore conducted a systematic review and meta-analysis of RCTs to compare the efficacy of ultrasound-guided technique with the traditional palpation for radial artery catheterization in pediatric patients.

## Materials and methods

This systematic review and meta-analysis was conducted according to the recommendations of the *Cochrane Handbook for Systematic Reviews of Interventions* and the guidelines established by the Preferred Reporting Items for Systematic Reviews and Meta-analyses (PRISMA) Group. Ethical approval and patient consent were not required in this study.

### Literature search

The electronic searches were performed by PubMed, Medline, Embase, Clinical Trial.gov registry, Cochrane Central Register of Controlled Trials (CCTR), and Cochrane Database of Systematic Reviews (CDSR) from their date of inception to December 2019. Medical Subject Headings (MeSH) terms and corresponding keywords were used for search with various combinations of the operators “AND” and “OR”: (MeSH exp. “Ultrasonography,” “Ultrasonics,” and keywords “ultrasonography*,” “ultrasonic*,” “ultrasound*,” and “ultrasound-guided”), (MeSH exp. “Radial Artery” and keywords “radial arteries,” “radial artery,” and “radial arterial”), and (MeSH exp. “Catheterization,” “Cannula,” “Catheter,” and keywords “catheterization,” “cannula,” “cannulation,” and “catheter”). We also checked the bibliographies of previous reviews and reviewed the reference lists of all retrieved articles for further identification of potentially relevant studies.

### Selection criteria

The inclusion criteria were as follows: (1) population: pediatric patients (age < 18 years) requiring radial arterial catheterization, (2) intervention: ultrasound-guided technique, (3) comparison: traditional palpation technique, and (4) study design: RCTs. We excluded abstracts, case reports, conference presentations, editorials, and reviews. For duplicate reports containing the same population data, only the one with the longest follow-up and most complete information was included.

### Data extraction and management

Two reviewers (W. Z. and K. L.) independently extracted the data from each article that met the inclusion criteria. The following data were recorded in a standardized form: name of the first author and published year, study period, country of study, age range, sample size, clinical setting, operator experience, ultrasound device, and ultrasound approach.

The primary outcomes included the rate of first-attempt and total success of radial arterial catheterization. The mean attempts to success, mean time to success, and incidence of complications were recorded as the secondary outcomes. Any discrepancy was resolved by thorough discussions.

### Assessment of risk of bias in included studies

Two authors (W. Z. and K. L.) assessed the risk of bias independently and in duplicate. We resolved disagreements by consensus or by consultation with a third review author (H.X.). The risk of bias was assessed according to the risk of bias tool of the Cochrane Collaboration. It included six domains: random sequence generation (selection bias); allocation concealment (selection bias); blinding of participants, providers, data collectors, outcome adjudicators, and data analysts (performance bias and detection bias); incomplete outcome data (attrition bias); selective outcome reporting (outcome reporting bias); and other biases. We defined trials as having “low,” “high,” or “unclear” risk of bias and evaluated individual bias items as described in the *Cochrane Handbook for Systematic Reviews of Interventions*.

### Statistical analysis

Review Manager version 5.3.5 (Cochrane Collaboration, Oxford, UK) was used for all data analysis. The relative ratio (RR) and weighted mean difference (WMD) were used to respectively analyze dichotomous outcome and continuous outcome. Both were reported with 95% confidence interval (CI), and a *P* value lower than 0.05 or a 95% CI that did not contain unity was considered statistically significant. Heterogeneity was evaluated with the *I*^2^ test, and the *I*^2^ > 50% indicated significant heterogeneity. In this meta-analysis, both fixed- and random-effect models were employed. Since similar results were obtained, only results of the random-effect model are presented.

## Results

### Literature search

**Fig. 1:**
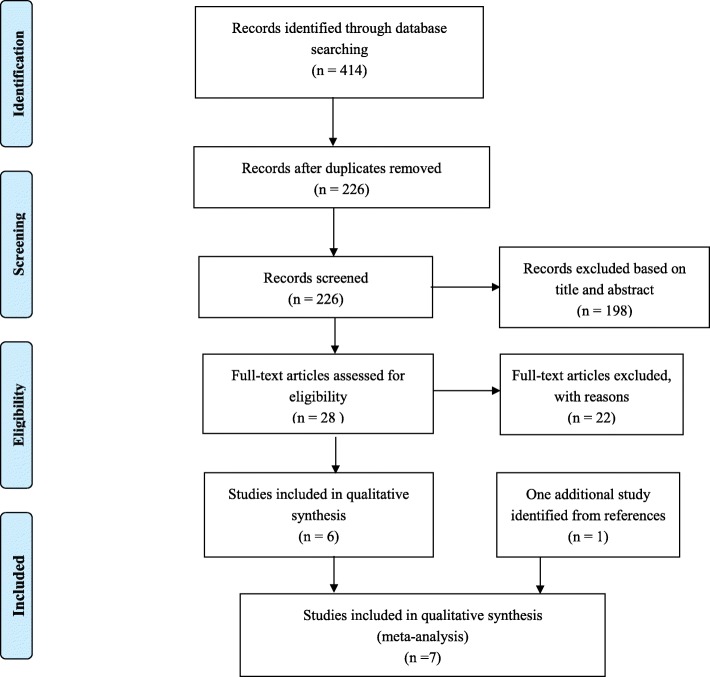
The procedure of literature search (flow diagram)

Two hundred eighty-six articles were identified from electronic databases (excluding duplicates). After application of the inclusion and exclusion criteria, seven studies [15–21] were finally included in this meta-analysis. All seven studies were randomized controlled trials of radial artery catheterizations. The literature search procedure is shown in Fig. 1. The seven included studies involved a total of 558 radial artery catheterizations, including 274 ultrasound-guided arterial catheterizations and 284 palpation catheterizations. The main characteristics of the included trials are summarized in Table 1.

**Table 1:** The characteristics of the included studies

First author (name, year)	Country	Age	Sample size	*N* (us-guided)	*N* (palpation)	Clinical setting	Operator experience	Ultrasound device	Ultrasound approach
Schwemmer 2006 [20]	Germany	Small children 6 m to 9 yr, average 40 ± 33 m	30	15	15	Operating room for major neurosurgery	More than 20 catheterizations	Sonos 5000 (Hewlett-Packard, Andover, MA, USA) 15 MHz	Short-axis out-of-plane
Ganesh 2009 [17]	USA	Elder children < 18 yr, 99.1 ± 69.3 m/99.6 ± 71.6 m	152	72	80	Operating room undergoing several surgeries	Fewer than 10 cases of us-guided technique	SonoSite 180plus (SonoSite, Bothell, WA, USA) 5–10 MHz	Short-axis out-of-plane
Ishii 2013 [16]	Japan	Small children and infants 18.4 m (7–28 m)	59	59	59	Operating room undergoing cardiac surgery	Familiar with the us-guided technique for catheterization	SonoSite 180 (SonoSite, Bothell, WA, USA) 2–7 MHz	Short-axis out-of-plane
Liu 2013 [21]	China	Small children 1–3 yr	60	30	30	Operating room undergoing surgery	Not reported	SonoSite 180 (SonoSite, Bothell, WA, USA) 6–13MHz	Not reported
Tan 2015 [19]	Singapore	Small children 6–24 m	40	20	20	Operating room undergoing surgery	Not reported	SonoSite 180 (SonoSite, Bothell, WA, USA) 6–13MHz	Not reported
Anantasit 2017 [18]	Thailand	Small children (20 m, 6–60 m or 32 m, 7–93 m)	84	41	43	Pediatric intensive care unit	More than 10 cases of both techniques	M-Turbo ultrasound system (SonoSite, Inc., Bothell, USA) 8–12 MHz	Short-axis
Min 2019 [15]	South Korea	Infants < 12 m	74	37	37	Operating room undergoing cardiac surgery	More than 50 cases of us-guided catheterization	Philips iE33 L15-7io (Philips Healthcare, Seattle, WA, USA) 7–15 MHz	Short-axis

*N* number of patients, *m* months, *yr* years, *us* ultrasound

### Risk of bias in included studies

**Fig. 2:**
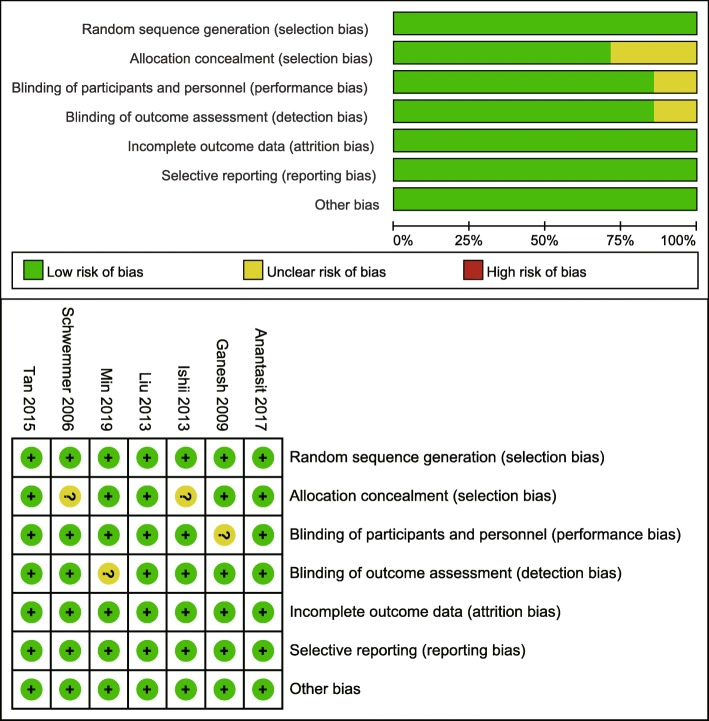
Assessment of risk

Figure 2 shows the risk of bias summary, which reflects judgments about each risk of bias item for each included study. Overall, three trials were categorized as at low risk of bias, four as unclear, and none as at high risk of bias. Adequate randomized sequence was generated in seven studies, and appropriate allocation concealment was reported in five trials. Blinding of outcome assessments was unclear or seldom reported in these seven trials, but the primary outcome was less prone to be influenced by the lack of blinding.

### Selective reporting

Six of the included studies reported success rate at the first attempt, and all of them gave the total success rate. Only four studies reported the second primary outcome—incidence of complications, and this might indicate selective reporting bias. The secondary outcome—mean time to success—was reported in all these studies, but only three trials showed mean ± standard deviation (SD) and another four did not demonstrate SD.

### Primary outcome: first-attempt success and total success

**Fig. 3:**
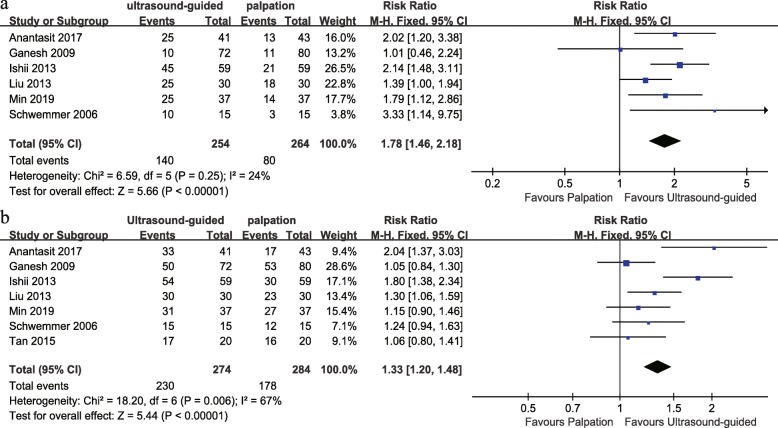
Forest plot comparing the rate of first-attempt success (**a**) and total success (**b**) for ultrasound-guided versus palpation

Six RCTs were used to calculate the pooled estimate for assessing the rate of first-attempt success. Overall, the rate of first-attempt success in the ultrasound-guided group and palpation group was 55.1% and 30.3%, respectively. Ultrasound-guided radial artery catheterization was associated with an increased first-attempt success (RR 1.78, 95% CI 1.46 to 2.18, *P* < 0.00001, Fig. 3), and no significant heterogeneity was shown among these studies (*I*^2^ = 24%).

The rate of total catheterization success was reported in all seven studies. The data demonstrated that the rate of total success was significantly higher in the ultrasound-guided group versus the palpation group (83.9% vs. 62.7%, RR, 1.33; 95% CI 1.20, 1.48; *P* < 0.00001, Fig. 3). However, significant heterogeneity was observed among the included studies for the total success (*I*^2^ = 67%, Fig. 3).

### Subgroup analysis based on age

**Fig. 4:**
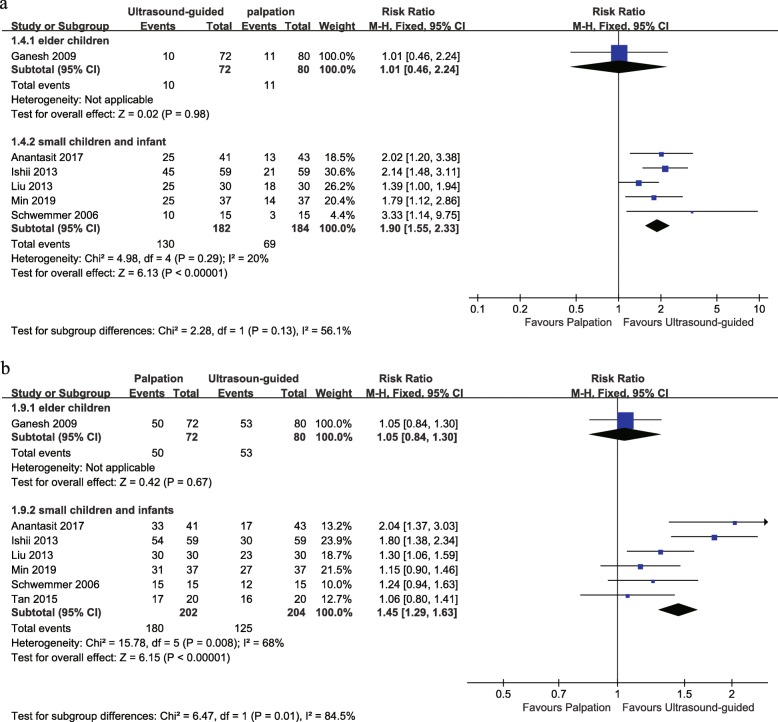
Forest plot comparing the rate of first-attempt success (**a**) and total success (**b**) for ultrasound-guided versus palpation based on age

There was only one trial reporting the data on the elder children. This study involved a wide age range (0–18 years), but most were elder children, with a mean age of 99 months in two groups. Other studies reported data on infants and small children. For the first-attempt success, no difference was detected between studies on elder children (one trial, RR 1.01, 95% CI 0.46 to 2.24), but a significant difference on small children and infants (five trials, RR 1.90, 95% CI 1.55 to 2.33). However, the test for subgroup effects revealed that age-related subgroup differences were not statistically significant (*P* = 0.13). In terms of the total success rate, there was also only one study on the elder children and no difference was shown (RR 1.05, 95% CI 0.84 to 1.30). Six trials reported the total success rate on small children and infants, and a significant difference was detected between studies (RR 1.45, 95% CI 1.29 to 1.63) (Fig. 4).

### Subgroup analysis based on the operator’s experience

**Fig. 5:**
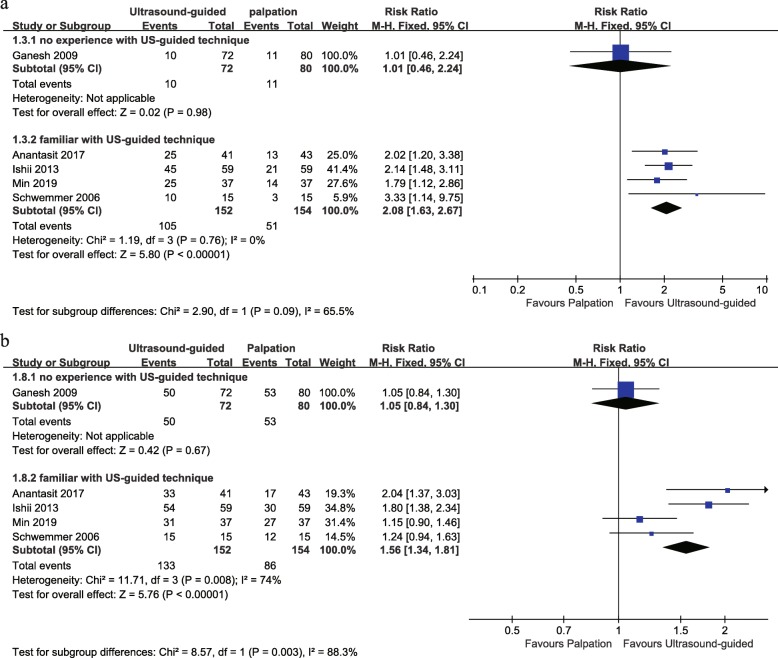
Forest plot comparing the rate of first-attempt success (**a**) and total success (**b**) for ultrasound-guided versus palpation based on experience

Of the seven studies included, five reported the operator’s experience on the radial arterial catheterization. Only one study reported that no operator had performed more than 10 ultrasound-guided arterial cannulations before this study, and the other studies had experience of more than 10 cases in arterial catheterization technique or familiar with the ultrasound-guided technique for central venous catheterization. Results showed that the ultrasound-guided technique did not significantly increase the success of catheterization at the first-attempt and the total success rate in the pediatric populations as compared with the palpation technique when the operator had minimal experience (one study, RR 1.01, 95% CI 0.46 to 2.24 for first-attempt success; RR 1.05, 95% CI 0.84 to 1.30 for total success). However, in the subgroup of studies in which operators had more experience, the success of catheterization at the first-attempt and the total success were both significantly increased in the ultrasound-guided group (four studies, RR 2.08, 95% CI 1.63 to 2.67 for first-attempt success; RR 1.56, 95% CI 1.34 to 1.81 for total success) (Fig. 5).

### Secondary outcomes

**Fig. 6:**
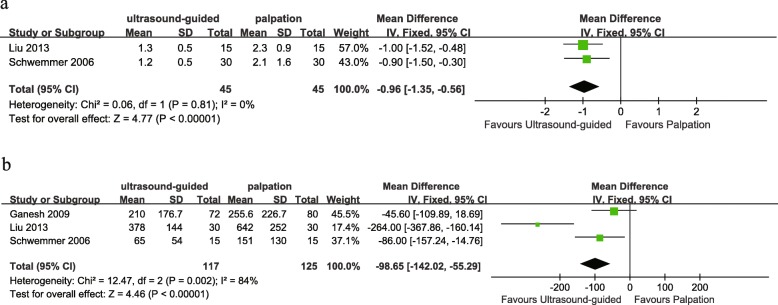
Forest plot comparing mean attempt to success (**a**) and mean time to success (**b**) for ultrasound-guided versus palpation

**Fig. 7:**
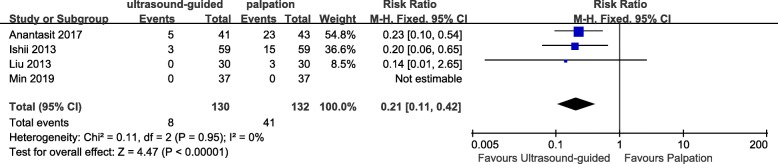
Forest plot comparing the incidence of hematoma for ultrasound-guided versus palpation

Similar to previous studies, ultrasound-guided radial artery catheterization was associated with less mean attempts to success (WMD − 0.96, 95% CI − 1.35 to − 0.56, *P* < 0.00001, Fig. 6), shorter mean time to success (WMD − 98.65 s, 95% CI − 142.02 to − 55.29, *P* < 0.00001, Fig. 6), and lower incidence of hematomas (RR 0.21, 95% CI 0.11 to 0.42, *P* < 0.00001, Fig. 7).

## Discussion

This is a further systematic review and meta-analysis of seven RCTs to evaluate the efficacy of the ultrasound-guided technique for radial arterial catheterization in pediatric populations. From the available data, the present meta-analysis showed that the ultrasound-guided technique was associated with a higher rate of first-attempt and total success in radial arterial catheterization for pediatric patients compared with the traditional palpation technique. Additionally, ultrasound-guided radial artery catheterization significantly reduced mean attempts to success, mean time to success, and incidence of the complication of hematoma.

Since the use of ultrasound guidance was first reported in arterial catheterization by Nagabhushan et al. [22] in 1976, ultrasound guidance has been increasingly used for arterial catheterization. Several reports were published to demonstrate the advantage of the ultrasound-guided technique for the insertion of an arterial catheter in adult populations [23, 24]. A recent meta-analysis conducted by Aouad-Maroun and his colleagues [14] aimed to compare the ultrasound-guided technique with other techniques (including the traditional palpation technique and Doppler) for arterial catheterization in pediatric patients. However, the low number of RCTs included in these studies made the evidence level relatively low. The high degree of heterogeneities was existed in these studies because of the inclusion of Ueda’s research comparing ultrasound with Doppler, which may lead to higher biases. Therefore, another meta-analysis is required to evaluate the curative effect of the ultrasound-guided technique versus the traditional palpation. This meta-analysis of comparative studies investigated the ultrasound-guided technique versus the traditional palpation technique for radial artery catheterization in pediatric populations.

The results of the present review confirmed previously reported advantages of the ultrasound-guided technique in pediatric patients. The use of ultrasound guidance for radial arterial catheterization could increase the rates of first-attempt and total success and reduce the incidence of complications. Hansen et al. [25] attributed that the ultrasound-guided technique could identify the target vessel, collateral vasculature, and nervous structures with real-time guidance of catheter insertion for arterial catheterization. Controversy remains on which is better, the short-axis out-of-plane technique or the long-axis in-plane technique, for radial arterial catheterization [26, 27]. Sethi et al. [26] found that the identification of the midpoint of the radial artery on a short-axis view was probably easier with the out-of-plane technique. This may explain why the short-axis was used in most of these studies included in our meta-analysis.

Technically, the operator’s experience plays an important role in using ultrasound guidance for radial arterial catheterization. Recent guidelines have recognized that ultrasound-guided cannulation rates are higher when trainees have developed general experience, skill, and dexterity [28]. The data from this present study suggested that ultrasound guidance significantly increased the first-attempt success rate when performed by an experienced operator (RR 1.98, 95% CI 1.04–3.77) [12, 19, 21] versus an inexperienced operator (RR 1.36, 95% CI 0.84–2.20). This was consistent with the previous report [29] that ultrasound guidance might be particularly useful in the most experienced operators for catheterization and inexperience may have prevented operators from realizing its full benefit.

Catheterization of the radial artery can be technically challenging in small children and infants due to the small vessel diameter, even for experienced operators, especially after repeated unsuccessful attempts causing complications such as hemorrhage and hematoma formation [30, 31]. In this meta-analysis, the results showed that ultrasound-guided radial artery catheterization in small children and infants could increase the rate of first-attempt success and total success when compared with the traditional palpation technique. There was only one study reporting about the elder children, and the data demonstrated that ultrasound guidance did not provide a higher success rate for the radial artery in elder children. However, the operators in this study were inexperienced and lacked training, which may influence the real effect of ultrasound guidance in the radial artery catheterization.

We further took the results of the mean attempts to success and mean time to success for assessing the effects of the ultrasound-guided radial artery catheterization. The results showed that the ultrasound-guided technique could also significantly reduce the mean attempts to success and mean time to success in radial arterial catheterization for pediatric populations compared to the traditional palpation technique.

As we know, a meta-analysis was a quantitative method that combined the data from several independent studies and researches on the same problem, pooling outcomes to achieve a more unbiased and scientific conclusion [32]. However, there were also several limitations existing in this present meta-analysis. First, the sample sizes were small in most of the included studies, which would decrease the overall precision of the estimates. Second, the RCTs in our meta-analysis were performed in different clinical settings and various patient groups, which may result in significant heterogeneity among the reviewed studies. Furthermore, other clinically relevant endpoints, such as patient pain and patient and physician satisfaction, were not assessed.

## Conclusion

The results of the current meta-analysis suggested that the ultrasound-guided technique was associated with higher rates of first-attempt and total success and lower incidence of hematoma compared with the traditional palpation technique. Ultrasound guidance is an effective and safe technique for radial artery catheterization, especially in small children and infants, and could be recommended to aid radial arterial catheterization. However, the results should be interpreted cautiously due to the heterogeneity among the studies.

## Data Availability

All the data supporting the conclusions of this article are included within the article.

## Abbreviations

RCT: Randomized controlled trial
CCTR: Cochrane Central Register of Controlled Trial
CDSR: Cochrane Database of Systematic Reviews
MeSH: Medical Subject Headings
RR: Relative ratio
WMD: Weighted mean difference
CIs: Confidence intervals
SD: Standard deviation
